# Natural history of X-linked hypohidrotic ectodermal dysplasia: a 5-year follow-up study

**DOI:** 10.1186/s13023-019-1288-x

**Published:** 2020-01-10

**Authors:** Sigrun Wohlfart, Ralph Meiller, Johanna Hammersen, Jung Park, Johannes Menzel-Severing, Volker O. Melichar, Kenneth Huttner, Ramsey Johnson, Florence Porte, Holm Schneider

**Affiliations:** 1 Center for Ectodermal Dysplasias & Department of Pediatrics, University Hospital Erlangen, Loschgestr. 15, 91054 Erlangen, Germany; 2 Department of Ophthalmology, University of Erlangen-Nürnberg, Erlangen, Germany; 3 Department of Ophthalmology, University of Düsseldorf, Düsseldorf, Germany; 4 Edimer Pharmaceuticals, Cambridge, USA; 5 EspeRare Foundation, Geneva, Switzerland

**Keywords:** Hypohidrotic ectodermal dysplasia, Natural history, Ectodysplasin A, Oligodontia, Dry eye, Heat intolerance

## Abstract

**Background:**

X-linked hypohidrotic ectodermal dysplasia (XLHED) is caused by pathogenic variants of the gene *EDA* disrupting the prenatal development of ectodermal derivatives. Cardinal symptoms are hypotrichosis, lack of teeth, and hypo- or anhidrosis, but the disease may also evoke other clinical problems. This study aimed at investigating the clinical course of XLHED in early childhood as the basis for an evaluation of the efficacy of potential treatments.

**Methods:**

25 children (19 boys and 6 girls between 11 and 35 months of age) with genetically confirmed XLHED were enrolled in a long-term natural history study. Clinical data were collected both retrospectively using parent questionnaires and medical records (pregnancy, birth, infancy) and prospectively until the age of 60 months. General development, dentition, sweating ability, ocular, respiratory, and skin involvement were assessed by standardized clinical examination and yearly quantitative surveys.

**Results:**

All male subjects suffered from persistent anhidrosis and heat intolerance, although a few sweat ducts were detected in some patients. Sweating ability of girls with XLHED ranged from strongly reduced to almost normal. In the male subjects, 1–12 deciduous teeth erupted and 0–8 tooth germs of the permanent dentition became detectable. Tooth numbers were higher but variable in the female group. Most affected boys had no more than three if any Meibomian glands per eyelid, most girls had fewer than 10. Many male subjects developed additional, sometimes severe health issues, such as obstructive airway conditions, chronic eczema, or dry eye disease. Adverse events included various XLHED-related infections, unexplained fever, allergic reactions, and retardation of psychomotor development.

**Conclusions:**

This first comprehensive study of the course of XLHED confirmed the early involvement of multiple organs, pointing to the need of early therapeutic intervention.

## Background

The term ectodermal dysplasia (ED) refers to a heterogeneous group of rare congenital conditions affecting the normal development of ectodermal structures including skin, teeth, hair, nails, and eccrine glands [1]. X-linked hypohidrotic ectodermal dysplasia (XLHED; MIM #305100), the most common form of ED, is characterized by a clinical triad of hypotrichosis, hypo-, oligo- or anodontia, and hypo- or anhidrosis [2]. The lack of sweat glands may lead to life-threatening hyperthermia which is mainly observed in early childhood [3]. The deficient development of other eccrine glands (salivary, lacrimal, sebaceous, submucosal, Meibomian, and mammary glands) entails recurrent respiratory infections, atrophic rhinitis, chronic skin issues, keratoconjunctivitis sicca, and, in case of females, breastfeeding difficulties [4–7].

XLHED is caused by variants of the X-chromosomal ectodysplasin A gene (*EDA;* NM_001399.4) leading to loss or dysfunction of the signaling protein EDA1 [8]. More than 200 different variants of this gene have been published so far, most of which are null mutations [9, 10]. A certain single-nucleotide polymorphism (SNP) rs3827760 (c.1109T4C; p.Val370Ala) in the gene *EDA1R*, a gain-of-function allele associated with hair thickness and shovel-shaped incisors that predominantly occurs in the Native American and East Asian population, was found to attenuate the severity of symptoms in at least one familial case of XLHED [11, 12].

More recently, our group reported successful prenatal treatment of XLHED in three boys with *EDA* null mutations by intra-amniotic administration of a replacement protein that induced the development of functional sweat glands, Meibomian glands and additional tooth germs [13]. The assessment of the natural history of a disease is indispensable for any evaluation of the efficacy of a potential treatment. In a series of exploratory studies conducted during the last 10 years, we captured patient-reported medical history data in subjects with genetically confirmed XLHED and tested minimally invasive methods for endpoint [14–20]. These cross-sectional studies have elucidated specific aspects of XLHED, provided new insights into the full spectrum of the phenotype and greater clarity about genotype-phenotype correlations. Validation of these findings, however, required the comprehensive collection and monitoring of clinical data over time, combined with repeated and objective endpoint assessments. Therefore, a long-term natural history study was initiated to compile systematically data on all XLHED-related clinical issues in untreated patients until the age of five years.

## Subjects and methods

### Study design and patients

19 male and 6 female patients between 11 and 35 months of age with genetically confirmed diagnosis of XLHED were enrolled in a long-term natural history study at our site (www.clinicaltrials.gov; NCT02099552) and completed this study. The protocol included the retrospective collection of clinical data using parent questionnaires and medical records, standardized systematic clinical examinations both at the time of enrolment and at the age of five years, repeated facial photographs, and yearly interrogations of the parents with a quantitative phone survey plus gathering of all relevant medical documents during the course of the study. Final data analysis was conducted at the age of 5 years when all deciduous teeth should have erupted and tooth germs of the permanent dentition except molars M3 (wisdom teeth) are expected to be calcified sufficiently to be detectable in panoramic radiographs.

Written informed consents of both parents (if available) to the participation of their child in this study were obtained. The study was approved by an independent institutional ethics committee and conducted according to national regulations and GCP/ICH guidelines. Subjects were included only if they had not received any investigational treatment prior to enrolment. Further exclusion criteria were congenital anomalies outside of those considered to be associated with XLHED, known hypersensitivity to pilocarpine or pilocarpine-like drugs, and implantable electronic devices.

### DNA analysis

Standard gene variant analysis including DNA extraction, polymerase chain reaction, and Sanger sequencing was performed as described previously [10]. Specific primer sequences and thermal cycling conditions for the detection of the XLHED-causing *EDA* mutations and screening for the polymorphism rs3827760 in *EDA1R* are available upon request.

### Anthropometric measurements and tooth quantification

Anthropometric measurements were obtained retrospectively from the child’s medical records or during the pediatric examinations. Body length in cm, weight in kg, and body mass index (BMI) in kg/m^2^ were compared with standard percentiles for the normal population [21]. Dentition was assessed by oral examinations and a panoramic radiograph at the age of five years. Primary teeth and tooth germs of the permanent dentition which are well distinguishable based on crown size, morphology, and root length in the radiographs were quantified by experienced dentists.

### Assessment of sweat duct density and sweat production

The number of plantar sweat ducts in a skin area of 36 mm^2^ was determined by confocal laser scanning microscopy with the VivaScope 1500 (Caliber Imaging & Diagnostics, New York, USA) and extrapolated to an area of 1 cm^2^. Sweating ability was assessed by quantification of pilocarpine-induced sweating (volumetry) in an area of 57 mm^2^ of the forearm for 30 min using the Wescor 3700 device (Wescor, Logan, USA) as described previously [13].

### Ophthalmic investigations

Symptoms of XLHED-related chronic dry eye were assessed by an experienced ophthalmologist as described previously. This included corneal examinations for signs of keratitis by staining of the ocular surface, Meibography (transillumination of the lower eyelids for the detection of Meibomian gland openings; normal range: 20 to 30) [22], measurement of the tear film break-up time (BUT) with a threshold of 10 s, determination of the ocular surface disease index (OSDI) score with a cutoff value of 12 and Schirmer’s test for the quantification of tear production (placement of a filter paper in the lower lid and measuring its moisturization during 5 min) with a cutoff value of 10 mm [16, 17, 23].

### Assessment of pulmonary function

Pulmonary function tests for the detection of asthma and bronchial obstruction included measurements of forced vital capacity (FVC), forced expiratory flow over 1 s (FEV1), and exhaled nitric oxide (eNO) level (Additional file 1). The results of body plethysmography were assessed by an experienced pediatric pneumologist.

### Skin examination

Potential skin issues were evaluated with quantitative patient surveys and the Eczema Area and Severity Index (EASI), a scoring system originally established for the assessment of extent and severity of atopic dermatitis. The EASI score for the whole body is the sum of scores for each of four defined body regions (head and neck, upper extremities, trunk, and lower extremities) and can range from 0 to a maximum 72 (0: free of symptoms; 1–5: mild; 6–22: moderate; 23–72: severe). The particular body scores were obtained by multiplying the sum of severity scores (0: none; 1: mild; 2: moderate; 3: severe) of the four key symptoms (erythema, infiltration, excoriations, and lichenification) with the area score (ranging from 0 to 6; representative for the percentage of affected area for each region) and by multiplying the result with the constant value for each body region proportionate to its percentage of the body surface [24–26].

### Quantitative patient survey and statistical analysis

The patients’ parents were asked once a year to fill in a questionnaire referring to different clinical and social aspects of XLHED. These surveys focused on the evaluation of the child’s sweating ability and (impaired) heat tolerance, XLHED-related issues with hair, teeth, eyes, nose and airways, voice, nutrition, skin, and nails. Continuous records of the various parameters including all data collected at the scheduled clinical examinations were analyzed. Box-and-whisker plots were used for the graphical depiction of the following statistical values: minimum, first quartile, median, third quartile and the maximum.

## Results

**Fig. 1:**
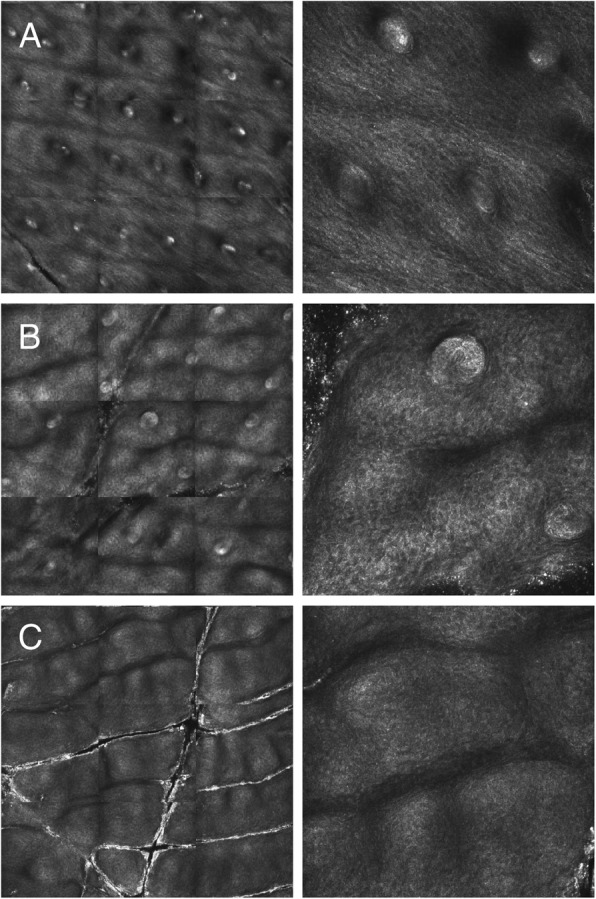
Plantar sweat duct densities in trizygotic triplets as determined by confocal laser scanning microscopy. Left: plantar area of 2.25 mm^2^. Right: magnification of a representative section. **a** female triplet without *EDA* mutation. **b** subject F3–004, heterozygous for the *EDA* variant c.467G > A. **c** subject M3–003, hemizygous for the indicated *EDA* variant

The *EDA* genotypes of all subjects are listed in Table 1. None of them carried the SNP rs3827760 in *EDA1R*. Parent-reported details on the medical history of each child (parent questionnaires) confirmed the known spectrum of XLHED symptoms; information from medical records was added if required (Additional file 2). Although a few rudimentary sweat pores could be detected in some boys (Table 1), anhidrosis was a common feature of all male subjects investigated in this study. Pilocarpine-induced sweating, the parameter most relevant with respect to heat tolerance, became evident only in female subjects (average sweat volume of 23.7 μl). Figure 1 visualizes the differences in sweat gland endowment between trizygotic triplets: a girl with the normal number and distribution of sweat ducts, her sister (subject F3–004, heterozygous carrier of the familial *EDA* variant) with clearly fewer sweat ducts, and their brother (subject M3–003, hemizygous) who does not seem to have sweat glands at all. Average body temperatures measured during clinical examinations at the study site (Table 1) were similar for girls and boys with XLHED, whereas heat intolerance indicated by hyperthermic episodes prior to enrolment was reported for all male subjects but for none of the girls in our cohort (Table 2). In 7 of 19 subjects (37%), unexplained fever led to hospitalization. Impaired thermoregulation had marked impact on daily life, outdoor sports, choice of vacation destinations, and the child’s ability to travel (Table 2).

**Table 1:** *EDA* genotype of the study participants and data related to their sweating ability

Code	Age at enrolment (months)	*EDA* variant	Changes at the amino acid level	Pilocarpine-induced sweat volume (μL)	Sweat pores/cm^2^ (plantar)	Body temperature (°C; average of independent measurements)
Male subjects						
M3–001	17	c.608C > T	p.Pro203Leu	0.0	22.2	37.15
M3–002	35	c.467_468del	p.Arg156GlnfsX2	0.0	1.4	36.90
M3–003	35	c.467G > A	p.Arg156His	0.0	0.0	36.95
M3–005	27	c.1075A > T	p.Lys359X	0.0	0.0	37.25
M3–007	24	c.463C > T	p.Arg155Cys	0.0	5.6	37.10
M3–008	11	c.1133C > T	p.Thr378Met	0.0	0.0	36.90
M3–009	33	c.1141G > C	p.Gly381Arg	0.0	0.0	37.15
M3–012	22	c.467G > A	p.Arg156His	0.0	0.0	37.15
M3–013	27	c.376_379del	p.Asp126ProfsX10	0.0	2.8	36.85
M3–014	33	c.793G > T	splice-site alteration	0.0	0.0	37.20
M3–015	17	c.925-3C > G	splice-site alteration	0.0	0.0	36.95
M3–016	21	c.502 + 1G > A	splice-site alteration	0.0	8.3	37.35
M3–017	28	c.463C > T	p.Arg155Cys	0.0	13.9	37.05
M3–018	29	c.467G > A	p.Arg156His	0.0	0.0	37.00
M3–019	26		duplication of exons 3–8	0.0	41.7	36.90
M3–020	31	c.1072C > T	p.Gln358X	0.0	0.0	36.50
M3–022	28	c.911A > G	p.Tyr304Cys	0.0	0.0	36.80
M3–023	17	c.648_665del18	p.Pro216_Gly221del	0.0	0.0	36.65
M3–025	26	c.1133C > T	p.Thr378Met	0.0	0.0	37.05
Average	25.63	/	/	0.00	5.05	36.99
SD	6.73	**/**	**/**	0.00	10.67	0.21
Female subjects						
F3–004	35	c.467G > A	p.Arg156His	23.0	625	37.20
F3–006	25	c.1112 T > A	p.Ile371Asn	9.0	u.a.	37.05
F3–010	35	c.572_589del	p.Pro191_Pro196del	16.0	u.a.	37.00
F3–011	31	c.659_676del	p.Pro220_Pro225del	17.0	850	37.20
F3–021	31	c.1072C > T	p.Gln358X	22.0	825	37.05
F3–024	21	c.546_581del36	p.Asn185_Pro196del	55.0	775	37.35
Average	29.67			23.67	768.75	37.14
SD	5.61			16.15	100.78	0.13

Abbreviations: *SD* standard deviation

**Table 2:** History regarding heat intolerance prior to enrolment

Code	Unexplained fevers	Associated seizures	Hospitalization due to heat intolerance	If yes, diagnosis	Reported heat intolerance	Impact of heat intolerance/reduced sweating on:			
						daily life	outdoor sports	choice of vacation destinations	ability to travel
Male subjects									
M3–001	Y	N	N	/	Y	N	N	Y	Y
M3–002	Y	N	Y	suspicion of pneumonia	Y	Y	N	Y	Y
M3–003	Y	N	N	/	Y	Y	N	Y	Y
M3–005	Y	N	Y	therapy-resistant hyperpyrexia	Y	Y	Y	Y	Y
M3–007	Y	N	N	/	Y	N	N	Y	Y
M3–008	Y	Y	Y	spasmodic laryngitis	Y	N	N	Y	Y
M3–009	Y	N	N	/	Y	N	N	Y	Y
M3–012	Y	N	Y	suspicion of respiratory tract infection	Y	N	N	Y	Y
M3–013	Y	N	N	/	Y	Y	N	Y	Y
M3–014	Y	N	N	/	Y	N	N	Y	Y
M3–015	Y	N	Y	hyperpyrexia	Y	Y	N	Y	Y
M3–016	N	N	N	/	Y	N	N	Y	Y
M3–017	Y	N	N	/	Y	Y	N	Y	Y
M3–018	Y	N	Y	fever, dehydration	Y	Y	Y	Y	Y
M3–019	Y	N	N	/	Y	N	Y	Y	Y
M3–020	Y	N	N	/	Y	Y	Y	Y	Y
M3–022	Y	N	N	/	Y	N	N	Y	Y
M3–023	Y	N	N	/	Y	N	N	Y	Y
M3–025	Y	N	Y	unexplained fever	Y	Y	Y	Y	Y
Female subjects									
F3–004	N	N	N	/	N	N	N	N	N
F3–006	N	N	N	/	N	N	N	N	N
F3–010	N	N	N	/	N	N	N	N	N
F3–011	N	N	N	/	N	N	N	N	N
F3–021	N	N	N	/	N	N	N	Y	N
F3–024	N	N	N	/	N	N	N	N	N

Abbreviations: *Y* yes; *N* no

**Fig. 2:**
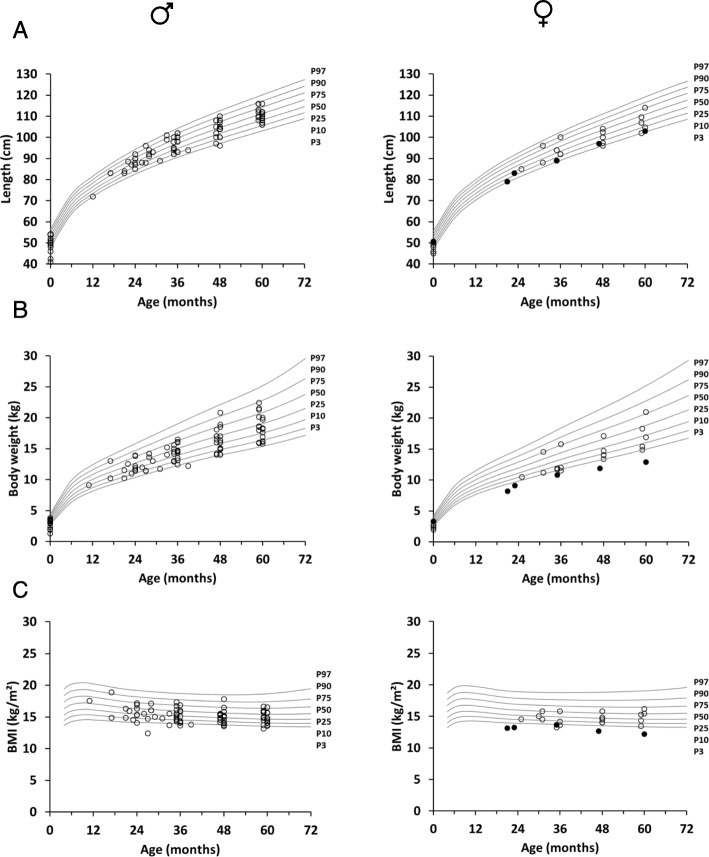
Growth charts of subjects with XLHED between 0 and 60 months of age in comparison with standardized percentiles for the normal population depicting **a** length-for-age (cm), **b** weight-for-age (kg), and **c** BMI-for-age (kg/m^2^) [21]. The filled circles represent a girl from Israel with normal birth weight but obvious growth retardation later on

Physical examination revealed the known ED-related abnormalities (Table 3). Body length, weight and resultant BMI-for-age charts between birth and 5 years of age (Fig. 2) were delineated to detect potential developmental delays. Most boys with XLHED grew between the 25th and the 75th percentiles (Fig. 2a). Four male subjects including three infants born prematurely had birth lengths below the third percentile, but only one of them fell below again at the age of 48 months. The girls showed normal growth, except for two pre-term babies with birth lengths below the third percentile (Fig. 2a). Body weights of most male and female subjects, however, did not reach the 50th percentile. Four of the boys (thereof three premature babies) and two preterm girls started with birth weights below the third percentile. One of the boys then showed clearly retarded weight gain and crossed the third percentile only at the age of 48 months (Fig. 2b). The weight of a female subject from Israel (born full-term with normal birth weight) fell below the third percentile and remained there until the last visit (Fig. 2b). The BMI-for-age chart reflects the slightly impaired weight gain of male and female subjects with XLHED: Five boys and three girls had a BMI below the P3 value at least at one time point during the 60 months (Fig. 2c). Head circumference and vital signs like blood pressure, heart rate, and respiratory rate were within the normal range in all subjects (data not shown).

**Table 3:** Physical exam findings

Detected abnormality	Number of affected individuals (%)	
	Male subjects	Female subjects
Hypo-, oligo- or anodontia (for more details see Table 4)	19 (100)	5 (83)
Lack or absence of eyebrows	19 (100)	5 (83)
Dry skin	19 (100)	1 (17)
Hypo- or atrichia	19 (100)	0 (0)
Sparse eyelashes	17 (89)	0 (0)
Hypoplastic jaws	16 (84)	1 (17)
Dysplastic or protruding ear(s)	16 (84)	0 (0)
Frontal bossing	16 (84)	0 (0)
Eklabium	12 (63)	1 (17)
Eczematous skin	10 (53)	1 (17)
Supernumerary nipple and/or nipples of different size	5 (26)	0 (0)
Initially retarded psychomotor development (later normal status)	4 (21)	0 (0)
Growth retardation	2 (11)	2 (33)
Conjunctivitis, inflamed lid margins	2 (11)	0 (0)

**Fig. 3:**
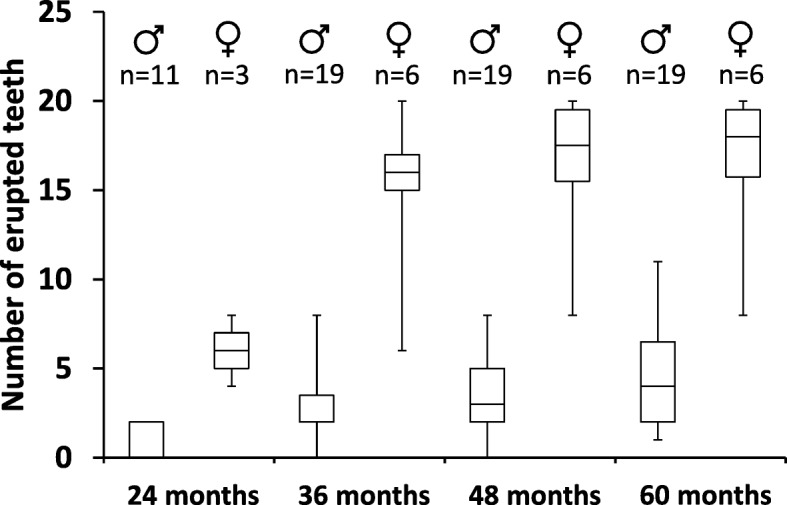
Number of erupted teeth in male and female subjects with XLHED at the age of 24, 36, 48 and 60 months

The number of erupted teeth was assessed annually between the age of 24 and 60 months, which confirmed the significantly impaired and delayed dentition in children with XLHED (Fig. 3). As expected, final tooth counts in girls with XLHED (Table 4) were more variable than in affected boys who got 4.8 teeth on average (1.9 and 1.7 in the upper quadrants, 0.6 in each of the lower quadrants) until their fifth birthday when 20 teeth (5 per quadrant) are present in the normal population. Female subjects like F3–006 with only 8 erupted teeth at the age of 60 months, however, indicate that the dentition in girls can be affected as severely as in boys with XLHED. Tooth germs of the permanent dentition were quantified on panoramic radiographs at the last study visit by experienced dentists (Table 4). In the male cohort, 8 germs of permanent teeth were the maximum detected (when 28 should be visible, excluding the third molars), but three boys did not even have a single tooth bud. The total number of tooth germs in the female subjects was 21.5 on average (SD 5.43) with more even distribution over the quadrants (Table 4).

**Table 4:** Number of deciduous teeth and tooth buds of the permanent dentition per quadrant

Code	Quadrant 1	Quadrant 2	Quadrant 3	Quadrant 4	Total number of teeth/tooth buds
Male subjects					
M3–001	2 + 1	2 + 1	1 + 0	0 + 0	5 + 2
M3–002	1 + 2	0 + 2	0 + 0	0 + 0	1 + 4
M3–003	1 + 2	1 + 2	0 + 2	0 + 1	2 + 7
M3–005	1 + 0	1 + 0	0 + 0	0 + 0	2 + 0
M3–007	3 + 2	3 + 2	3 + 1	3 + 1	12 + 6
M3–008	3 + 3	3 + 3	1 + 1	1 + 1	8 + 8
M3–009	2 + 2	2 + 1	1 + 1	1 + 1	6 + 5
M3–012	2 + 1	2 + 0	0 + 0	0 + 0	4 + 1
M3–013	1 + 2	1 + 2	0 + 1	0 + 1	2 + 6
M3–014	3 + 2	3 + 2	0 + 1	1 + 1	7 + 6
M3–015	3 + 1	1 + 1	0 + 0	0 + 0	4 + 2
M3–016	3 + 2	3 + 2	0 + 1	0 + 1	6 + 6
M3–017	3 + 2	3 + 3	2 + 0	2 + 0	10 + 5
M3–018	1 + 2	1 + 2	0 + 0	0 + 1	2 + 5
M3–019	1 + 0	1 + 1	0 + 0	0 + 0	2 + 1
M3–020	2 + 2	2 + 2	1 + 1	1 + 1	6 + 6
M3–022	2 + 0	2 + 0	0 + 0	0 + 0	4 + 0
M3–023	1 + 0	1 + 0	1 + 1	1 + 1	4 + 2
M3–025	1 + 0	1 + 0	1 + 0	1 + 0	4 + 0
Average	1.90 + 1.37	1.74 + 1.37	0.58 + 0.53	0.58 + 0.53	4.79 + 3.79
SD	0.88 + 0.96	0.93 + 1.01	0.84 + 0.61	0.84 + 0.51	2.94 + 2.64
Female subjects					
F3–004	4 + 5	4 + 5	5 + 6	5 + 6	18 + 22
F3–006	3 + 5	1 + 3	1 + 2	3 + 3	8 + 13
F3–010	5 + 6	5 + 6	5 + 7	5 + 7	20 + 26
F3–011	4 + 5	3 + 3	3 + 5	5 + 5	15 + 18
F3–021	5 + 7	5 + 7	5 + 7	5 + 7	20 + 28
F3–024	4 + 4	4 + 4	5 + 7	5 + 7	18 + 22
Average	4.17 + 5.33	3.67 + 4.67	4.00 + 5.67	4.67 + 5.83	16.5 + 21.5
SD	0.75 + 1.03	1.51 + 1.63	1.67 + 1.97	0.82 + 1.60	4.55 + 5.43

**Fig. 4:**
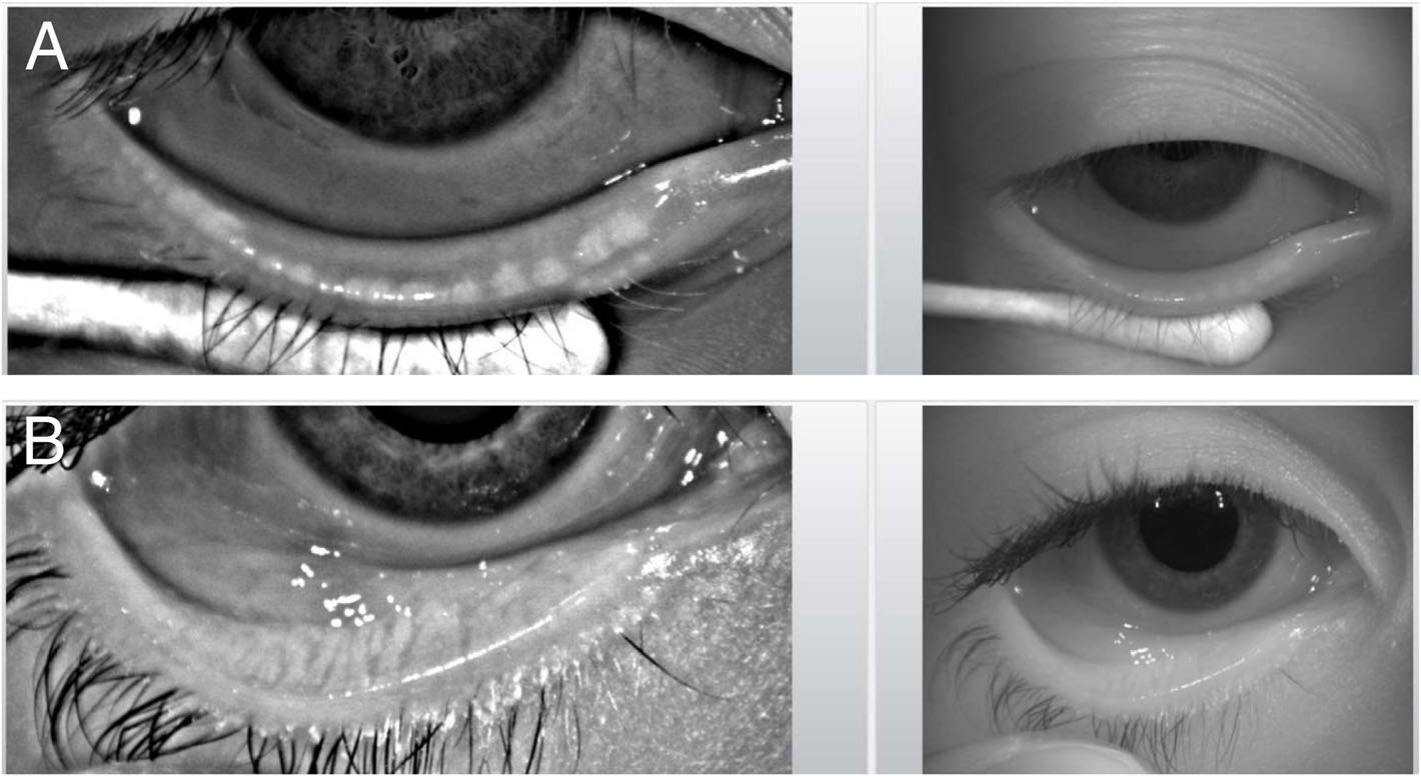
**a** Infrared images of the everted lower eyelid highlight some rudimentary Meibomian gland ducts (white dots) in a representative male subject with XLHED. **b** more than 20 fully developed Meibomian gland ducts, extending from the lower tarsus to the orifices at the lid margin, in an age-matched healthy control

Ophthalmic investigations revealed bilateral superficial punctate keratitis, a consequence of chronic dry eye, in 7 of 18 boys with XLHED (39%) but in none of the 6 girls (Table 5). All male subjects showed a significant lack or complete absence of Meibomian glands, the sole producers of the lipid components that stabilize the tear film on the ocular surface. Where more than 6 Meibomian gland openings per lower eyelid were detected (normal range: 20–30, median 26) [27], they appeared incompletely developed (Fig. 4). The number of Meibomian glands was reduced also in 4 of the 6 girls (67%).

**Table 5:** Ophthalmic assessments

Code	Corneal examination	Meibomian gland openings^a^		BUT (s)		OSDI score	Schirmer’s test (mm)	
		Right eye	Left eye	Right eye	Left eye		Right eye	Left eye
Male subjects								
M3–001	SPK (on both sides)	1	2	3	3	11.1	12	10
M3–002	N	0	0	3	2	0.0	5	6
M3–003	SPK (on both sides)	2	3	2	4	8.3	u.a.	u.a.
M3–005	N	3	1	5	4	5.0	11	5
M3–007	N	0	0	10	12	2.5	u.a.	u.a.
M3–008	SPK (on both sides)	2	3	1	2	13.8	20	u.a.
M3–009	N	5	4	4	4	5.0	10	4
M3–012	N	0	0	u.a.	u.a.	5.0	12	17
M3–013	SPK (on both sides)	1	3	5	5	11.1	6	11
M3–014	N	1	1	10	11	5.0	7	4
M3–015	u.a.	0	3	5	6	8.3	u.a.	u.a.
M3–016	SPK (on both sides)	0	0	8	10	5.5	u.a.	u.a.
M3–017	N	0	2	12	12	2.7	14	> 25
M3–018	SPK (on both sides)	1	2	4	4	19.4	u.a.	u.a.
M3–019	N	6^b^	9^b^	7	u.a.	2.7	16	10
M3–020	SPK (on both sides)	1	0	u.a.	u.a.	22.2	u.a.	u.a.
M3–022	N	1	1	u.a.	u.a.	5.0	u.a.	u.a.
M3–023	N	1	0	u.a.	u.a.	5.0	u.a.	u.a.
M3–025	N	6	6	u.a.	u.a.	2.7	> 25	> 25
Average	/	1.39	1.72	5.64	6.08	7.38	/	/
SD	/	1.72	1.71	3.30	3.77	5.85	/	/
Female subjects								
F3–004	N	> 20	> 20	14	14	0.0	u.a.	u.a.
F3–006	N	9	8	14	12	11.1	u.a.	u.a.
F3–010	N	8	5	8	6	2.5	4	u.a.
F3–011	N	> 20	> 20	9	8	2.5	10	5
F3–021	N	6	5	12	10	0.0	> 25	> 25
F3–024	N	5	5	u.a.	u.a.	0.0	u.a.	u.a.

Abbreviations: ^a^, maximum number detected in the lower lid during two independent examinations; N, normal; SPK, superficial punctate keratitis; u.a., unable to assess; ^b^, not fully developed

Sufficient tear film stability as indicated by a normal break-up time (BUT) on both eyes was observed only in three male subjects (Table 5); 11 of 14 boys (79%) presented with diminished BUT values, and Ocular Surface Disease Index (OSDI) scores above the threshold of 12 suggested an early development of dry eye syndrome already in three of them. Schirmer’s test, which could not be conducted on both eyes in all affected boys, still revealed normal values in 5 of 10 subjects (50%). Two of the girls showed reduced BUT, but all had normal OSDI scores (Table 5).

Any adverse events (AEs) including serious adverse events (SAEs) that occurred during this study were documented thoroughly. In our cohort of male subjects with XLHED, 85 AEs (thereof 17 SAEs) were recorded, between 0 and 6 per year and individual, most of them XLHED-related infections (Table 6). The frequency and distribution of AEs was similar in the female subjects.

**Table 6:** Adverse events (AEs) / serious adverse events (SAEs) during the observation period

	Male subjects			Female subjects		
Event	Number of subjects (%)	AEs	Thereof SAEs	Number of subjects (%)	AEs	Thereof SAEs
1. XLHED-related infections						
Respiratory	14 (74)	25	7	3 (50)	5	0
Ear	4 (21)	15	3	1 (17)	6	0
Skin	2 (11)	5	0	2 (33)	4	0
Nose	4 (21)	4	0	1 (17)	1	0
Eye	1 (5)	1	0	1 (17)	1	0
Other	0 (0)	0	0	1 (17)	3	0
2. XLHED-unrelated infections	5 (26)	7	0	2 (33)	4	0
3. Unexplained fevers						
with seizure	1 (5)	2	2	0 (0)	0	0
without seizure	1 (5)	1	0	1 (17)	1	0
4. Allergic reactions	5 (26)	8	2	1 (17)	1	0
5. Developmental retardation	5 (26)	6	0	1 (17)	2	0
6. Other	6 (32)	11	3	1 (17)	5	2

The annual phone visits and completed parent questionnaires also highlighted recurrent respiratory and skin problems. Upper airway infections were the most common issue; 22 children had to struggle with such problems (both sexes) and four of them (male patients) developed asthma. Furthermore, 22 children suffered from hoarseness of the voice (irrespective of sex). With the exception of two girls, all subjects were afflicted with dry skin, 15 of them also with eczema or atopic dermatitis. Every male but no female subject was reported to have a constantly blocked or running nose. Nosebleeds occurred frequently; 11 subjects had more than ten nosebleeds per year (ranging up to 36 events). In three children with eczematous skin lesions, the EASI scoring system was used and a maximum score of 10.1 was observed. Body plethysmography of 22 subjects and evaluation of their lung function parameters indicated obstructive airway conditions in seven boys with XLHED but in none of the female subjects.

## Discussion

So far only one large collection of ED-related clinical issues has been published, the web-based Ectodermal Dysplasia International Registry (EDIR). It summarizes patient-reported medical data from 141 male and 82 female patients of different age groups (mean ages of 17.8 and 32.1 years, respectively). Self-assessment revealed life-long relevant health problems, not restricted to the typical symptom triad but also regarding growth, skin, nails, respiratory, and ocular issues [28]. The natural history data reported here broaden the current knowledge about the clinical course of XLHED by focusing on the most critical first years of life when life-threatening hyperthermic events and complications of disease usually occur [3]. A higher degree of awareness among medical personnel, mainly pediatric dentists and pediatricians, would probably accelerate proper clinical diagnosis and thereby prevent heat-associated complications. In this respect, the parent-reported major impact of heat intolerance on different aspects of daily life, which was probably underappreciated in previous studies, seems to be of particular relevance. Our study confirms an ED-associated increased risk of growth retardation that was reported by others [29], although persistent undernourishment and developmental delay were not common in our cohort. Nevertheless, most of our patients were situated in the lower half of the growth charts, and it is conceivable that children growing up under conditions of poor nutrition or medical care (e.g. no provision of prostheses allowing proper mastication) are at higher risk of developmental retardation.

As expected, our female subjects with XLHED showed milder phenotypes than affected boys, but none of them was asymptomatic. Although females with XLHED used to be considered only as carriers in numerous publications, it has become well known that most of them are actually affected by XLHED-related symptoms like hypotrichosis, hypodontia, and conically shaped teeth (carrier detection is possible in at least 70% of cases) [30]. Additional XLHED-related issues, such as deficient breast development and its consequences (breastfeeding difficulties and psychosocial problems), cannot be assessed before puberty but have been found to be frequent among female carriers of *EDA* mutations [18]. Therefore, future medical treatment might also be considered for female patients. Phenotypic heterogeneity, however, was another feature of our female cohort, while genotype-phenotype correlation is rather strong in boys with XLHED [14, 20]. The latter may not be reflected fully by our randomly recruited cohort in which the minority of male subjects with hypomorphic mutations is not represented, but only solid natural history data may allow to predict the severity of XLHED for each known *EDA* variant. Skewed X-chromosome inactivation is likely to explain phenotypic differences between females with similar *EDA* variants and has been discussed in a few case reports [31–33].

Although this natural history study was completed in 2018, medical care of the patients at our center will continue, enabling further data collection until adulthood. Due to the sparse availability of data for rare diseases like XLHED, those studies may not only be valuable for genotype-based prediction of the course of disease and attempts to prevent complications, but also serve as comparators allowing the evaluation of future drug therapies [13, 34–36]. Considering the prospective nature, the duration of this study, and the number of patients followed, our data will be relevant for regulatory agencies who evaluate outcomes from open-label drug trials in subjects with XLHED.

## Conclusions

This first comprehensive natural history study characterized the course of XLHED in male and female patients during the first five years of life, confirming the early involvement of multiple organs and pointing to the need of early therapeutic intervention. The data emphasize that null mutations in EDA consistently result in anhidrosis and severe heat intolerance, while the phenotypes of individuals who carry such mutations heterozygously show a remarkable variability. Although female subjects with one normal X chromosome are often less severely affected, they are not asymptomatic and should receive more attention by researchers in the field. Besides the classic symptom triad of XLHED, patients usually suffer from a number of additional clinical issues, such as skin, eye and respiratory problems that may also be amenable to early treatment.

## Supplementary information

**Additional file 1.** Pulmonary Function Test (selected parameters). FVC (L), FEV1 (L) and eNO level (ppb).

**Additional file 2.** ED-related medical history. Reported abnormalities.

## Data Availability

The datasets used and analysed during the current study are available from the corresponding author on reasonable request.

## Abbreviations

BUT: Tear film break-up time
EASI: Eczema Area and Severity Index
ED: Ectodermal dysplasia
EDA: Ectodysplasin A
eNO: Exhaled nitric oxide
FEV1: Forced expiratory volume
FVC: Forced vital capacity
HED: Hypohidrotic ectodermal dysplasia
OSDI: Ocular Surface Disease Index
SNP: Single-nucleotide polymorphism
SPK: Superficial punctate keratitis
XLHED: X-linked hypohidrotic ectodermal dysplasia
