# Supplementary material for: Natural history of X-linked hypohidrotic ectodermal dysplasia: a 5-year follow-up study
Source: Orphanet J Rare Dis. 2020 Jan 10;15:7. doi: 10.1186/s13023-019-1288-x (PMC6954509; doi:10.1186/s13023-019-1288-x)
Supplement: Supplementary file 1 — Additional file 1. Pulmonary Function Test (selected parameters). FVC (L), FEV1 (L) and eNO level (ppb). [file 13023_2019_1288_MOESM1_ESM.docx]

**Additional file 1. Pulmonary Function Test (selected parameters)**

| **ID** | **FVC (L)** | **FEV1 (L)** | **eNO level (ppb)** |
| --- | --- | --- | --- |
| **Male patients** | | | |
| M1 | 1.38 | 1.23 | 7.5 |
| M2 | 1.18 | 1.01 | n.d. |
| M3 | 0.97 | 0.95 | n.d. |
| M5 | 1.18 | 1.01 | 9.0 |
| M7 | 1.31 | 1.27 | 3.0 |
| M8 | 1.21 | 1.14 | n.d. |
| M9 | 1.50 | 1.43 | 8.0 |
| M12 | 0.95 | 0.76 | n.d. |
| M13 | 1.43 | 1.23 | n.d. |
| M14 | 1.41 | 1.09 | n.d. |
| M15 | n.d. | n.d. | n.d. |
| M16 | 1.31 | 1.08 | 1.0 |
| M17 | 1.35 | 1.26 | n.d. |
| M18 | 1.72 | 1.46 | 2.0 |
| M19 | 1.39 | 1.27 | n.d. |
| M20 | 1.03 | 0.92 | n.d. |
| M22 | 1.41 | 1.34 | n.d. |
| M23 | n.d. | n.d. | n.d. |
| M25 | 1.19 | n.d. | 22.5 |
| **Female patients** | | | |
| F4 | 1.18 | 1.08 | n.d. |
| F6 | 1.04 | 0.97 | n.d. |
| F10 | 0.92 | 0.91 | n.d. |
| F11 | 1.07 | 1.00 | n.d. |
| F21 | 1.26 | 1.24 | 18.5 |
