# Supplementary material for: Natural history of X-linked hypohidrotic ectodermal dysplasia: a 5-year follow-up study
Source: Orphanet J Rare Dis. 2020 Jan 10;15:7. doi: 10.1186/s13023-019-1288-x (PMC6954509; doi:10.1186/s13023-019-1288-x)
Supplement: Supplementary file 2 — Additional file 2. ED-related medical history. Reported abnormalities. [file 13023_2019_1288_MOESM2_ESM.docx]

**Additional file 2. ED-related medical history**

| **Reported abnormality** | **Number of subjects (%)** | |
| --- | --- | --- |
|  | **Male patients** | **Female patients** |
| Hypo-, oligo- or anodontia | 19 (100) | 5 (83) |
| Hyperthermic episodes | 16 (84) | 0 (0) |
| Eczematous skin | 13(68) | 0 (0) |
| Repeated hoarseness | 10 (53) | 0 (0) |
| Frequent nosebleeds | 8 (42) | 0 (0) |
| Severe airway infections | 7 (37) | 0 (0) |
| Dry eye disease | 5 (26) | 0 (0) |
| Preterm birth | 3 (16) | 2 (33) |
| Growth retardation | 3 (16) | 0 (0) |
| Frequent eye infections | 3 (16) | 0 (0) |
| Retarded psychomotor development | 2 (11) | 0 (0) |
